# Label- and Reagent-Free Optical Sensor for Absorption-Based Detection of Urea Concentration in Water Solutions

**DOI:** 10.3390/s24092754

**Published:** 2024-04-26

**Authors:** Carlo Anelli, Vanessa Pellicorio, Valentina Bello, Sabina Merlo

**Affiliations:** 1 Department of Electrical, Computer and Biomedical Engineering, University of Pavia, Via Ferrata 5, 27100 Pavia, Italy; carlo.anelli01@universitadipavia.it (C.A.); valentina.bello@unipv.it (V.B.); 2 Department of Drug Sciences, University of Pavia, Viale Taramelli 12, 27100 Pavia, Italy; vanessa.pellicorio@unipv.it

**Keywords:** optical sensing, absorption measurements, label-free sensor, urea, LED, photodiode, thermopile

## Abstract

Contactless and label-free detection of urea content in aqueous solutions is of great interest in chemical, biomedical, industrial, and automotive applications. In this work, we demonstrate a compact and low-cost instrumental configuration for label-free, reagent-free, and contactless detection of urea dissolved in water, which exploits the absorption properties of urea in the near-infrared wavelength region. The intensity of the radiation transmitted through the fluid under test, contained in a rectangle hollow glass tubing with an optical pathlength of 1 mm, is detected in two spectral bands. Two low-cost, low-power LEDs with emission spectra centered at λ = 1450 nm and λ = 2350 nm are used as readout sources. The photodetector is positioned on the other side of the tubing, in front of the LEDs. The detection performances of a photodiode and of a thermal optical power detector have been compared, exploiting different approaches for LED driving current modulation and photodetected signal processing. The implemented detection system has been tested on urea–water solutions with urea concentrations from 0 up to 525 mg/mL as well as on two samples of commercial diesel exhaust fluid (“AdBlue™”). Considering the transmitted intensity in presence of the urea–water solution, at λ = 1450 nm and λ = 2350 nm, normalized to the transmitted intensity in presence of water, we demonstrate that their ratio is linearly related to urea concentration on a wide range and with good sensitivity.

## 1. Introduction

Contactless and label-free detection of urea content in aqueous solutions is of great interest in chemical, biomedical, industrial, and automotive applications. For example, urea detection is quite important in clinical applications for the monitoring of hemodialytic therapy [1,2]. Another increasingly important application of urea sensors is related to the current main societal issue that is air pollution. Diesel vehicles crossing our cities emit polluting gases such as nitrous oxides NOx, formed by nitric oxide (NO) and nitrogen dioxide (NO_2_). Selective catalytic reduction (SCR) technology was developed to reduce these harmful emissions; it is based on the addition of a diesel exhaust fluid (DEF), also known by the registered trademark “AdBlue™”, to the exhaust gases to convert NO_x_ into nitrogen gas and aqueous vapors. According to the ISO standard ISO 22241-1:2019 [3], an efficient DEF consists ideally of 32.5% by weight of urea and 67.5% by weight of deionized water. Hence, concentration of the urea must always be in the correct range to ensure adequate emission reduction. Urea concentration control in AdBlue™ is then fundamental since, as time goes by, it can undergo changes because of water evaporation or fluid contamination.

Most of the commercially available systems for the control of the suitable concentration of urea in DEF samples are based on refractometric techniques; a drop of fluid is deposited upon a glass prism that is crossed by a laser beam (usually at the wavelength of approximately 590 nm) and the refractive index (RI) of the sample is determined by exploiting light refraction. However, it is well known that RI measurements are “non-specific”, meaning that alterations in DEF with substances that do not affect the refractive index or that are added to compensate for the reduction in index due to dilutions would not be identified. For this reason, measurements of urea content in DEF with commercial refractometers requires subtraction of the RI contribution due to the biuret, which can represent an issue in correct urea determination in heat-treated (i.e., exhaust) AdBlue™ [4]. Moreover, commercial refractometers need to spill out the liquid to perform the measurement and they are not suitable for in-line continuous measurements.

Other DEF quality sensors were reported in the literature for measuring urea concentration, based on optical principles [5,6], electrical factors [7], and ultrasonic measurements [8].

In [9], a platinum thin-film sensor in combination with the 3ω method was applied to determine the concentration of urea in DEF with a resolution of 1% by weight. The 3ω method is a thermal method to measure thermal conductivity and heat capacity. Since the thermal parameters of water and urea are different, the authors were able to distinguish between water and AdBlue™ using the 3ω method. The probe needs to be inserted in the fluid under test. A lock-in amplifier-based technique was necessary to detect the amplitude and phase of the 3ω voltage signal as a function of the driving frequency and its dependence on the urea concentration.

In [6], Kumawat et al. proposed a refractometric optical sensor based on differential interferometry constituted by a flow cell realized with microfabrication techniques (photolithography and wet etching). The bottom of the resulting sample cell is characterized by a periodical structure with regions of different height. Light emitted by a He-Ne laser and modulated at 1.5 kHz is shined onto the periodic flow cell which reflects light by acting as a diffraction grating. Light intensity diffracted at the zeroth and first orders are recorded with two photodiodes. By computing the ratio between the two measured intensity when dilutions of a DEF fluid with different RI are injected in the flow cell, the authors could demonstrate that the obtained normalized signal depends on the DEF fluid concentration, that can be thus monitored with a precision of ±1% and a lower limit of detection of approximately 0.0045%.

Another optical method, based on liquid chromatography, was proposed in [5] to determine urea content in exhaust DEF. The proposed system, which exploits the urea absorption peak in the UV region (specifically at 190 nm), is bulky and expensive and its accuracy could be limited by the presence of small amounts of other compounds (such as isocyanic acid, cyanuric acid, and acetic acid).

Fendri et al. presented a work based on electrochemical impedance spectroscopy to characterize AdBlue™ impedance as a function of the frequency (from 40 Hz to 110 MHz) [7]. Experimental results show how the shape of the Nyquist plot depends on AdBlue™ concentration and temperature.

In [8], Gurusamy et al. developed a system for detecting the concentration of urea in DEF. The design involves a piezoelectric ultrasound transmitter and a receiver; the measurement can be performed either by measuring the time of flight of ultrasound waves between transmitter and receiver or by measuring the amplitude of the received signal in terms of frequency using a voltage-to-frequency converter. Experimental results confirm that a 1% change in concentration of urea in diesel exhaust fluid can be detected by the measurement system.

A wide variety of assays rely on the use of chemical agents to recognize the analyte of interest and to react with the analyte to produce specific color changes, in particular, for biological applications. For example, in urea sensors, the most exploited element is urease, an enzyme that catalyzes the hydrolysis of urea, generating ammonium and bicarbonate ions [10,11,12]. Several urea biosensors have been also reported in recent reviews on this topic [13,14,15,16].

However, chemical reagents as well as labeling markers are expensive and harmful, creating further problems for their safe disposal. Moreover, since DEF is corrosive to iron, copper, bronze, and some aluminum alloys, contact-less detection systems able to work remotely would be highly preferable.

Near-infrared (NIR) spectroscopy offers an appealing approach to chemical analyses [17]. Optical sensing in the NIR can successfully exploit the intrinsic absorption patterns that provide the basis to quantify the constituents. A distinguish feature of spectral absorption measurements is that they can be label- and reagent-free as well as contact-less. Although NIR-based analytical methods may successfully rely on the absorption fingerprints of a variety of substances, it is still quite challenging to apply them in compact sensing systems for analyzing water-based mixtures since water is, per se, highly absorbing in several, wide wavelength regions of the NIR spectrum. Approaches based on multiwavelength intensity measurements are potentially attractive to keep into account, and even compensate for, the simultaneous effect of water.

In a previous work, we demonstrated a mixed fiberoptic and free-space instrumental configuration, combining three short-wave infra-red (SWIR) LEDs and two InGaAs amplified photodiodes for contact-less fluidic sensing by measuring light intensity transmittance across a channel microslide. The functionality of the system was tested on urea–water solutions with urea concentrations only up to 200 mg/mL [18].

Here, we present a more compact setup specific for the detection of urea concentration in urea–water solutions in a much wider range of concentrations, up to 525 mg/mL. It has been also used to analyze two samples of commercial diesel exhaust fluid (DEF), known as AdBlue™. The instrumental configuration for label-free, reagent-free, and contactless detection of urea dissolved in water exploits the absorption properties of urea in near infrared and compensates for water absorption in the same ranges. The intensity of the radiation transmitted through the fluid under test, contained in a rectangle hollow glass tubing with an optical pathlength of 1 mm, is detected. Two low-cost LEDs with emission spectra centered at 1450 nm and 2350 nm and the peak output power of a few mW are used as readout sources, placed almost in contact with the 1-centimeter-wide flat side of the 5-centimeter-long tubing. The photodetector is positioned on the other side of the tubing, in front of the LEDs. The total distance between LED and detector is less than 1 cm, to minimize optical losses. The detection performances of an amplified InGaAS photodiode and of a thermal optical power detector based on a thermopile have been compared, exploiting different approaches for the LED driving current modulation and photodetected signal processing. The implemented detection system has been tested on urea–water solutions with urea concentrations from 0 up to 525 mg/mL (up to ~34.4% weight of urea), a wide range of interest for several industrial and automotive applications, as well as used to analyze two samples of commercially available AdBlue™, with a nominal composition of 32.5% in weight of urea and 67.5% in weight of demineralized water. Transmittance of light generated by the LED emitting around λ = 2350 nm is strongly reduced by the absorption increment due to an increasing fraction of urea in the solution. This occurs because the absorption coefficient of urea around λ = 2350 nm is much higher than that of water [19]. Since even water (solvent of all tested solutions) absorbs in the region around λ = 2350 nm, its contribution was accounted and compensated for by measuring the transmittance in the wavelength range around λ = 1450 nm, where water, and not urea, exhibits a strong absorption band. Considering *T1450*(*C*) and *T2350*(*C*) as the transmissivities in presence of the urea–water solutions, at λ = 1450 nm and λ = 2350 nm, respectively, obtained by normalizing the transmitted intensity in presence of urea mixtures to the transmitted intensity in presence of pure water, we have demonstrated their ratio *R*(*C*) = *T1450*(*C*)/*T2350*(*C*), as a significant output variable specific for urea detection. We achieved a sensitivity *S* = Δ*R*(*C*)/Δ*C*∼2.5 (g/mL)^−1^ with good linearity in a wide range of urea concentrations.

## 2. Materials and Methods

### 2.1. Instrumental Configuration

**Figure 1:**
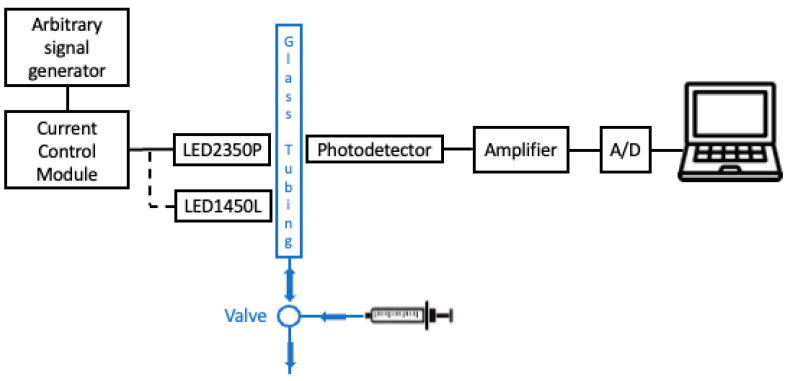
Instrumental configuration for optical, label-free urea detection.

The instrumental configuration for label- and reagent-free optical detection of urea in aqueous solutions is reported in Figure 1. A rectangle hollow glass tubing, 5 cm long and 1 cm wide, providing an optical pathlength for absorption measurements of 1 mm, was filled with urea–water solutions at different concentrations one at a time. Each sample was discarded after measurements by pushing air through the tubing. Two low-cost LEDs with emission in the near infrared were used as readout sources, placed almost in contact to one flat side of the tubing. LED1450 (LED1450L by Thorlabs, Newton, NJ, USA) is an InGaAsP/InP LED mounted on a TO-18 package with a spherical glass lens. On a spectral band typically centered at λ = 1450 nm and with FWHM = 105 nm, it emits an optical power of 5 mW when continuously driven at 50 mA. LED2350 (LED2350P by Thorlabs, Newton, NJ, USA) has a parabolic reflector and, on a spectral band typically centered at λ = 2350 nm (±50 nm) and FWHM = 220 nm, it emits an optical power of ~0.8 mW when driven in quasi-CW mode, that is modulated ON–OFF at 2 kHz with a peak current of 200 mA. Both LEDs were secured in a L-shaped mount (LEDMF-Ø1/2” by Thorlabs, Newton, NJ, USA) attached to a three-axis, manual linear translation stage. The LEDs were driven ON–OFF with a Laser Diode Current Control Module (LDC8005 by Thorlabs, Newton, NJ, USA) by means of an arbitrary signal generator (33500B Waveform Generator by KEYSIGHT, Colorado Springs, CO, USA) connected to the modulation input of the controller.

The photodetector for measuring the transmitted optical power though the sample was positioned on the other side of the tubing, in front of the LEDs. We used a manual linear x-y-z stage to align the detector with LED sources. To perform the optimal alignment, LEDs were moved, until reaching the position that yielded the highest intensity of transmitted light, determined as the maximum peak-to-peak voltage signal amplitude monitored on an oscilloscope when water was filling the channel. The total distance between LEDs and detector was maintained shorter than 1 cm, to minimize optical losses. We tested and compared the performances of two different detectors: an amplified indium–gallium–arsenide (InGaAS) photodiode (DET10D2 by Thorlabs, Newton, NJ, USA) and a thermal optical power detector based on a thermopile (TD4XP by Thorlabs, Newton, NJ, USA) attached to a metal-core printed circuit board (PCB), mounted on a heat sink.

The effective photodetected contribution due to absorption is provided by the output voltage difference with LED ON and LED OFF. Different ON–OFF current modulation patterns were applied to match the different response time of the photodetectors. When using the amplified photodiode, the driving current of both LEDs was modulated ON–OFF at a frequency of 2 kHz, with 50% duty cycle, setting a peak current of 50 mA for LED1450 and of 190 mA for LED2350, as suggested by the technical specifications. When using the thermopile, the driving current of LED1450 was modulated ON–OFF at a frequency of 250 mHz, with a 50% duty cycle, applying a peak current of 50 mA. Since LED2350 cannot be operated in DC, the driving current function was the product of the ON–OFF modulations at 250 mHz and at 2 kHz, with peak current of 190 mA.

The amplified, analog output signals provided by the photodetectors were acquired by a laptop computer by means of an USB-connected, analog-to-digital converter board (Analog Discovery 2 by Digilent, Pullman, WA, USA). The photodetected signal provided by the photodiode when turning on the LED2350 was further amplified by an AC-coupled, non-inverting operational amplifier with a gain of 100. The photodetected signal provided by the thermopile was amplified by a two-stage, DC-coupled, non-inverting operational amplifier with a total gain of 10^6^ when using the LED2350 and 10^4^ when using the LED1450.

With the photodiode, for each fluid sample, we performed three acquisitions at a sampling rate of 400 kHz, each one lasting 11 ms, thus containing approximately 20 periods of the 2 kHz signal. On the other hand, when using the thermopile, for each fluid sample, we performed a single acquisition at a sampling rate of 100 Hz, lasting 60 s, thus containing approximately 14 periods of the 250 mHz signal. Since the response time of the thermopile is approximately 1.5 s, the output voltage in the “ON” half-period of the 250 mHz signal is proportional to the average of the 2 kHz component. The acquired signals were analyzed and processed off-line with dedicated scripts in a MATLAB environment.

### 2.2. Sample Preparation

Samples with different concentrations of urea powder in deionized water were prepared and tested. Urea (grade purity 99.5%) used as standard was provided by Merck KGaA (Darmstadt, Germany). Water was obtained from the Millipore Direct-QTM system (Merk–Millipore, Milan, Italy). In a first experiment, we tested fluid samples with the following concentrations (*C* in mg/mL): 0, 100, 150, 200, 250, 300, 350, and 400 (corresponding to 0, 9.1, 13.0, 16.7, 20.0, 23.1, 25.9, and 28.6%g/g by weight of urea). In a second experiment, we tested fluid samples with the following concentrations (*C* in mg/mL): 0, 400, 425, 450, 475, 500, and 525 (corresponding to 0, 28.6, 29.8, 31.0, 32.2, 33.3, and 34.4%g/g by weight of urea), which is the range that includes the nominal urea content in AdBlue™ that consists of 32.5% by weight of urea and 67.5% by weight of deionized water, equivalent to a urea–water solution with *C*~481 mg/mL. Two commercial samples, namely DEF1 and DEF2, of AdBlue™ purchased from gas stations were tested without any kind of preprocessing or conditioning.

Refractive index measurements were performed on a few samples of urea–water solutions and on samples of DEF1 and DEF2 with a commercial digital refractometer (PCE-DRB1 by PCE Instruments, Southampton, UK) to roughly estimate the urea concentration with an additional method, used in commercial detection systems, to be compared with the estimates of the optical method.

The urea concentration in DEFs was also investigated with high-performance liquid chromatography (HPLC), considered as a reference measurement method. An HPLC Agilent 1200 system (Waldbronn, Germany) equipped with a mobile-phase online degasser, a quaternary pump, and a diode array detector (DAD) was used. Analyses were performed on a Gemini^®^ C18 analytical column (150 × 2.0 mm i.d., 5 μm, Phenomenex, Torrance, CA, USA) at 0.2 mL/min flow, with an injection volume of 20 μL and a stop time of 10 min. A total of 100% MilliQ water was used as the mobile phase. The column temperature was set at 30 °C. UV-Vis spectra were acquired in the 100–600 nm range, and chromatograms were recorded at 200 nm. The ChemStation software C.01.07 SR3 was used for data acquisition and processing. For the calibration curve, urea was dissolved in distilled water to obtain concentrations of 50, 100, 250, 500, and 800 µg/mL. AdBlue™ samples were diluted 1:1000 with distilled water before injection. The analyses were carried out in triplicate for each sample.

## 3. Results

### 3.1. Refractive Index Measurements on the Tested Samples

Refractive index (RI) measurements of the tested samples were performed with a commercial standard refractometer. Table 1 summarizes the measured RI values of three samples of urea–water solutions with *C* = 450 mg/mL, 475 mg/mL, and 500 mg/mL and of the commercial samples DEF1 and DEF2 of diesel exhaust fluid, purchased from gas stations. Measurements were performed at room temperature (T = 21.5 ± 0.5 °C). Typical values (nD20) reported in the literature for AdBlue™, at T = 20 °C, are in the range 1.3814–1.3843 [20]. Following a procedure also discussed in [20], by linearly fitting the data of RI for urea–water solutions reported in Table 1, we estimated for our samples C_DEF1 = 463.2 mg/mL (31.66%g/g in weight of urea) and C_DEF2 = 471.2 mg/mL (32.03%g/g in weight of urea). Sample testing with HPLC yielded C_DEF1 = 490.1 ± 2.3 mg/mL (corresponding to 32.89 ± 0.23%g/g) and C_DEF2 = 489.3 ± 5.6 mg/mL (corresponding to 32.85 ± 0.56%g/g).

### 3.2. Experimental Results with the Photodiode

**Figure 2:**
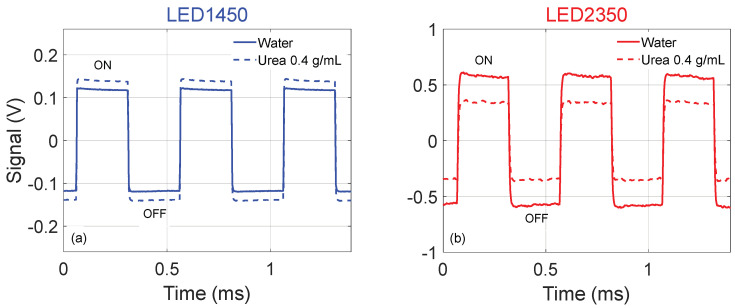
Examples of the 2 kHz signals provided by the photodiode (**a**) using LED1450 and when water is filling the tubing (solid line) and when urea–water solution at *C* = 0.4 g/mL is filling the tubing (dash line); (**b**) using LED2350 and when water is filling the tubing (solid line) and when urea–water solution at *C* = 0.4 g/mL is filling the tubing (dash line).

Examples of the 2 kHz signals provided by the photodiode are reported in Figure 2. Figure 2a shows a few periods of the acquired signal when using LED1450 and water is filling the tubing (solid line) and when the urea–water solution at *C* = 0.4 g/mL is filling the tubing (dash line). By comparing the traces in Figure 2a, it is possible to observe that the peak-to-peak amplitude is larger when urea is present in the sample, since an increasing amount of urea in the solution corresponds to a decreasing fraction of water, which is a strong absorber at λ = 1450 nm (while urea is not), thus lowering absorption.

Figure 2b shows a few periods when using LED2350 and water is filling the tubing (solid line) and when the urea–water solution at *C* = 0.4 g/mL is filling the tubing (dash line). By comparing the traces in Figure 2b, it is possible to observe that the peak amplitude is larger when pure water is present. For increasing concentrations of urea, absorption increases, transmittance decreases, and the signal amplitude diminishes.

For each acquisition, we calculated the RMS (root–mean–square) value on 20 periods, whereas the average peak amplitude of the acquired signals was determined considering 13 periods, further normalized to the values recorded in presence of water, considered as a reference fluid. As a matter of fact, the transmitted intensity *T1450*(*C*) and *T2350*(*C*), normalized to the transmissivity in presence of pure water, is linearly proportional to both considered output parameters (RMS and average amplitude).

**Figure 3:**
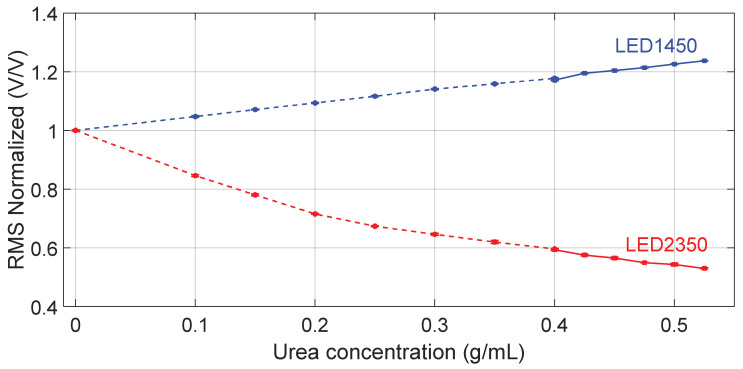
RMS values of the acquired signals with both LEDs and photodiode, normalized to the RMS value obtained when water is filling the channel as reference fluid, as a function of the tested concentrations. Circle markers ●, ● and the dashed line refer to data collected in the first experiment, covering the urea concentration range 0–0.400 g/mL, whereas square markers ■, ■ and the solid line refer to the second experiment, covering the urea concentration range 0.400–0.525 g/mL. Markers in blue refer to LED1450 whereas markers in red refer to LED2350. The error bar around the markers represents the average value ± standard deviation.

Figure 3 shows the RMS values, normalized to the RMS value obtained when water is filling the channel as a reference fluid, as a function of the tested concentrations, obtained at two wavelengths.

**Figure 4:**
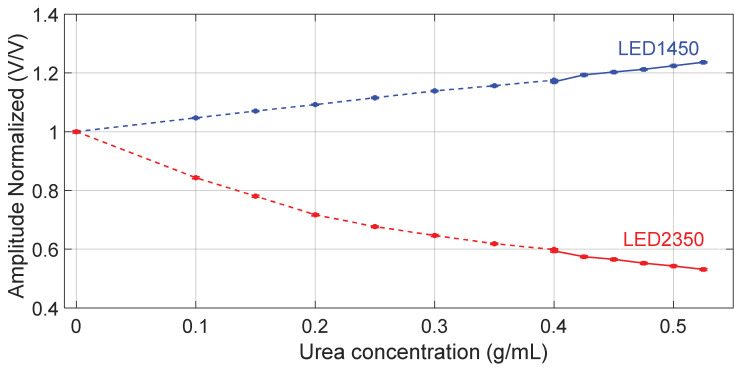
Amplitude of the acquired signals with both LEDs, normalized to the value obtained when water is filling the channel as reference fluid, as a function of the tested concentrations. Circle markers ●, ● and the dashed line refer to data collected in the first experiment, covering the urea concentration range 0–0.400 g/mL, whereas square markers ■, ■ and the solid line refer to the second experiment, covering the urea concentration range 0.400–0.525 g/mL. Markers in blue refer to LED1450 whereas markers in red refer to LED2350. The error bar around the markers represents the average value ± standard deviation.

Figure 4 shows the average peak amplitude, normalized to the value obtained when water is filling the channel as a reference fluid, as a function of the tested concentrations, measured at both wavelengths. In both Figure 3 and Figure 4, data points indicated with circle markers and a dashed line refer to the outcome of the first experiment, covering the urea concentration range 0–0.4 g/mL, whereas data points indicated with square markers and a solid line refer to the results of the second experiment, covering the urea concentration range 0.400–0.525 g/mL. Markers in blue refer to LED1450 whereas red markers refer to LED2350.

**Figure 5:**
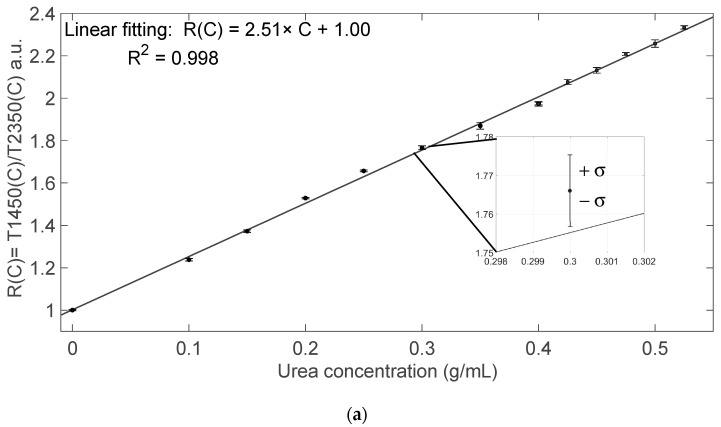

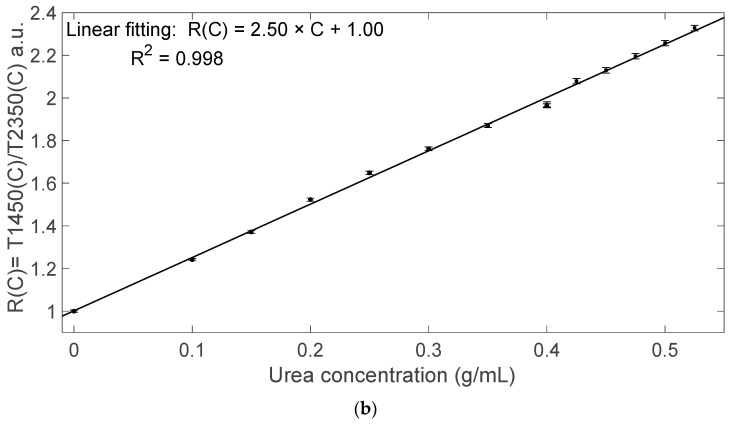
Transmitted intensity ratio *R*(*C*) = *T1450*(*C*)/*T2350*(*C*) as a function of the urea concentration. (**a**) Ratio calculated between the RMS values shown in Figure 3; (**b**) ratio calculated between the amplitude values shown in Figure 4. Circle markers ● refer to data collected in the first experiment, covering the urea concentration range 0–0.400 g/mL, whereas square markers ■ refer to the second experiment, covering the urea concentration range 0.400–0.525 g/mL. Black solid line: linear fitting. The error bar around the markers represents the average value ± standard deviation.

Finally, we calculated the ratio *R(C)* = *T1450*(*C*)/*T2350*(*C*) that is reported in Figure 5 as a function of urea concentration *C* in g/mL. This ratio, either calculated between the RMS values (Figure 5a) or between the peak amplitude (Figure 5b), can be considered specific for urea detection. Linear fitting of all data leads to a sensitivity *S =* Δ*R*(*C*)/Δ*C*~2.5 (g/mL)^−1^ and limit of detection LoD = 3 × σ*/S* ≈ 5 mg/mL ≈ 0.5%g/g, where σ is the standard deviation of the ratio *R* calculated on transmitted intensity analysis in presence of a water sample that is the reference fluid.

### 3.3. Estimate of Urea Concentration in DEF Samples Using Data Collected with the Photodiode

With the photodiode, for each DEF sample, we acquired three times the transmitted signals at both wavelengths. Using the linear equations reported in Figure 5a as calibration curves, we estimated the urea concentration and the relative results with uncertainties are reported in Table 2. Using the linear calibration curve of Figure 5b, we estimated the urea concentration values reported in Table 3 with uncertainties. The estimated values fall within the expected range of concentration for AdBlue™, that is 31.8–33.2%g/g by weight of urea, or approximately 466–497 mg/mL [20]. The average relative uncertainty of the estimated concentrations is around 9.12 × 10^−3^.

### 3.4. Experimental Results with the Thermopile

**Figure 6:**
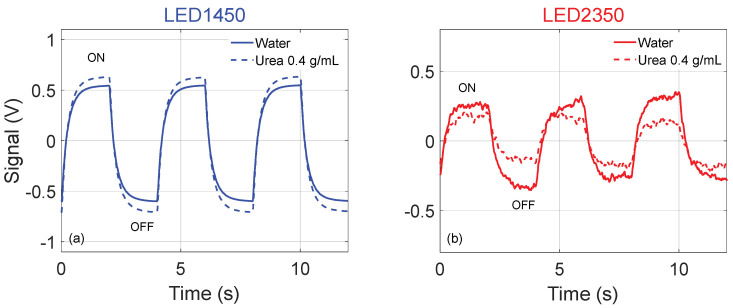
Examples of the 250 mHz signals provided by the thermopile (**a**) using LED1450 and when water is filling the tubing (solid line) and when urea–water solution at *C* = 0.4 g/mL is filling the tubing (dash line); (**b**) using LED2350 and when water is filling the tubing (solid line) and when urea–water solution at *C* = 0.4 g/mL is filling the tubing (dash line).

Examples of the 250 mHz signals provided by the thermopile are reported in Figure 6. Figure 6a shows three periods of an acquired signal when using LED1450 and water is filling the tubing (solid line) and when the urea–water solution at *C* = 0.4 g/mL is filling the tubing (dash line). By comparing the traces in Figure 6a, it is possible to observe that the peak-to-peak amplitude is slightly larger when urea is present, since an increasing amount of urea in the solution is related to a lower water absorption and thus a larger transmitted intensity.

Figure 6b shows three periods of an acquired signal when using LED2350 and water is filling the tubing (solid line) and when the urea–water solution at *C* = 0.4 g/mL is filling the tubing (dash line). By comparing the traces in Figure 6b, it is possible to observe that the peak amplitude is larger when pure water is present. This behavior is quite similar to what was already observed and discussed for the signals provided by the photodiode.

**Figure 7:**
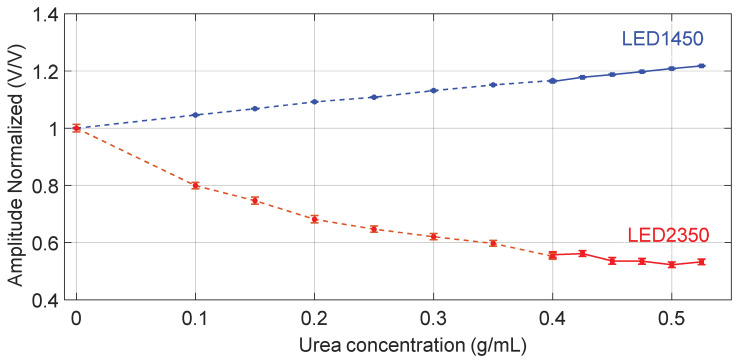
Amplitude of the acquired signals with both LEDs and thermopile, normalized to the value obtained when water is filling the channel as reference fluid, as a function of the tested concentrations. Circle markers ●, ● and the dashed line refer to data collected in the first experiment, covering the urea concentration range 0–0.400 g/mL, whereas square markers ■, ■ and the solid line refer to the second experiment, covering the urea concentration range 0.400–0.525 g/mL Markers in blue refer to LED1450 whereas markers in red refer to LED2350. The error bar around the markers represents the average value ± standard deviation.

For each acquisition, we calculated the average peak amplitude of the acquired signals, considering 13 periods, further normalized to the values recorded in presence of water, considered as a reference fluid. Since a temporal drift was observed in the photodetected signal provided by the thermopile when using LED2350, the RMS value could not be considered as a significant parameter. So, in this case, we calculated (on one acquisition for both wavelengths) the average amplitude, normalized to the value obtained when water is filling the channel as a reference fluid, i.e., *T1450*(*C*) and *T2350*(*C*), shown in Figure 7 as a function of the tested concentrations.

**Figure 8:**
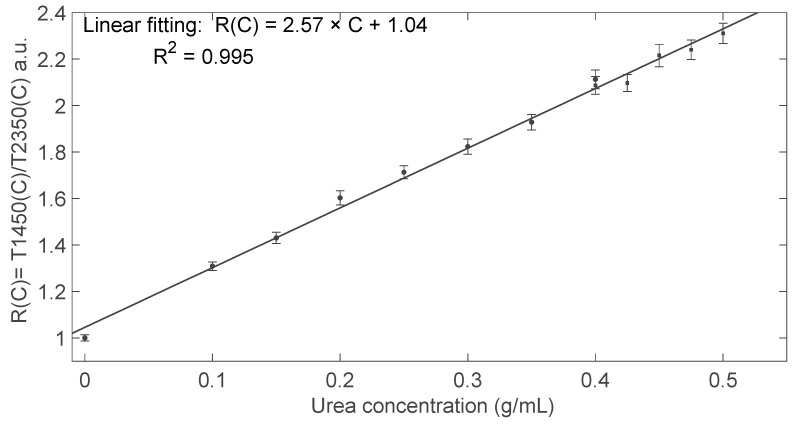
Transmitted intensity ratio *R*(*C*) = *T1450*(*C*)/*T2350*(*C*) as a function of the urea concentration, calculated between the amplitude values shown in Figure 7. Circle markers ● refer to data collected in the first experiment, covering the urea concentration range 0–0.400 g/mL, whereas square markers ■ refer to the second experiment, covering the urea concentration range 0.400–0.525 g/mL. Black solid line: linear fitting. The error bar around the markers represents the average value ± standard deviation.

Finally, we calculated the ratio *R*(*C*) = *T1450*(*C*)/*T2350*(*C*) that is reported in Figure 8 as a function of urea concentration *C*. This ratio was calculated between the normalized transmitted amplitudes shown in Figure 7 and can be considered a specific parameter for urea detection. Linear fitting of the data suggests a sensitivity *S =* Δ*R*(*C*)/Δ*C*~2.57 (g/mL)^−1^, thus slightly higher but in substantial agreement with that found with the photodiode, and limit detection LoD = 3 × σ/*S* ≈ 15 mg/mL ≈ 1.5%g/g, where σ is the standard deviation of the ratio *R* calculated on transmitted intensity analysis in the presence of a water sample that is the reference fluid. Results collected with LED2350 as the readout source and using the thermopile as the detector are less significant for concentrations higher than 0.5 g/mL because the enhanced absorption by urea strongly reduces the transmitted intensity and the low thermopile sensitivity does not ensure a sufficient signal-to-noise ratio.

### 3.5. Estimate of Urea Concentration in DEF Samples Using Data Collected with the Thermopile

Finally, we acquired the transmitted signals at both wavelengths in presence of both DEFs in the tubing using the thermopile. Using the linear equation reported in Figure 8 as a calibration curve, we then estimated the urea concentration in both samples. The results with uncertainties are reported in Table 4. The estimated values fall within the expected range of concentration for AdBlue™. The average relative uncertainty of the estimated concentrations is around 53.2 × 10^−3^.

## 4. Discussion and Conclusions

Precise measurement of the urea content in DEF is extremely important, since, according to the ISO standard ISO 22241-1:2019, urea concentration must be in the correct range in order to ensure the correct operation of selective catalytic reduction (SCR) systems in diesel engines and adequate reduction of NOx gas emission. In this work, we have presented a compact sensing setup based on NIR spectroscopy for the specific detection of urea concentration in a urea–water solution in a wide range of concentrations, from 0 to 525 mg/mL. We have exploited the implemented system to analyze two samples of DEF. The measurement configuration features two LEDs sources (emitting in spectral regions around λ = 1450 nm and λ = 2350 nm) and glass tubing; for light detection, a photodiode and a thermopile were tested and compared. Both measuring systems were calibrated with urea–water solutions, showing a sensitivity around 2.5 (g/mL)^−1^, and allowed us to measure the mean values of the urea content in DEFs in agreement with values measured with the standard HPLC reference methods. A direct comparison of the results obtained with the different methods is reported in Table 5. However, while the configuration with the photodiode ensured uncertainty values of the same order of magnitude of those obtained with HPLC (in the range 0.14–0.56%g/g), the standard deviations measured with the thermopile are slightly larger (~1.8%g/g). When comparing the performances of the photodetectors, it must be highlighted that the amplified photodiode allows for faster measurements even in the presence of ambient light but requires DC polarization and good alignment with the light source. On the other hand, the thermopile acts as a voltage generator with a large active area and thus easier positioning, without requiring any DC power supply in the sensing region but can be affected by surrounding VIS/MIR radiation and by sources of thermal power and heat. Thermopiles have a very slim shape, which becomes an important feature for applications where space is tight. Eventually, it is interesting to compare the proposed spectroscopy-based techniques with the HPLC standard and the refractometric method used in commercial devices for DEF quality control. HPLC is extremely sensitive and reliable, but it requires expensive instrumentation and sample pretreatment such as dilutions by a factor of 1000. Indeed, with HPLC, it is preferable to measure urea concentrations of the order of few µg/mL, to avoid column overload. Refractometric urea sensors surely require more simple measurement steps, but they are non-specific; furthermore, a drop of liquid needs to be spilled out to perform the analysis. Other quality sensors, like those cited in the Introduction, have some disadvantages. In fact, in [6], a refractometric optical sensor is proposed with the issue of being “non-specific”. Moreover, to generate the highly coherent readout light beam, a He-Ne laser, much bulkier than the LEDs, is necessary. Another optical method, based on liquid chromatography, is proposed in [5]. This system, which exploits the urea absorption peak in the UV region (specifically at 190 nm), is bulky and expensive; moreover, it is well known that UV light can be dangerous and must be used with great care. In [7], a contact sensor based on electrochemical impedance spectroscopy is proposed. It requires, to perform measurements on a wide frequency range (from 40 Hz to 110 MHz), an electrical spectrum analyzer that is expensive and not suitable for a compact or portable sensor. In [8], the authors have proposed an ultrasonic sensor, but its resolution is limited since it cannot detect changes in concentration of urea in DEF lower than 1%. In [9], the authors have reported a platinum thin-film sensor in combination with the 3ω method to determine the concentration of urea in DEF with a resolution of only 1% by weight of urea. The probe must be inserted in the fluid under test, so it is a contact sensor. Our optical configurations overcome many of these drawbacks; indeed, they allow urea detection over a wide concentration range (0–525 mg/mL) with high linearity. Also, the technique based on NIR spectroscopy is highly specific for urea detection and label-free at the same time. Moreover, thanks to the presence of the glass tubing, the measurement could also be performed in-line and in real time, simply by inserting a shunt path and without spilling the liquid. Eventually, the proposed configurations could also be exploited for other applications where measurements of urea content in a wide range of concentrations need to be performed with a low-cost, simple method.

## Figures and Tables

**Table 1:** Mean RI values of three samples of urea–water solutions and of DEF1 and DEF2, measured with a commercial refractometer.

Sample	RI
*C* = 450 mg/mL	1.3807
*C* = 475 mg/mL	1.3823
*C* = 500 mg/mL	1.3842
DEF1	1.3815
DEF2	1.3821

**Table 2:** Estimated mean and uncertainty values of urea concentration in the commercial samples DEF1 and DEF2 using the linear equations reported in Figure 5a as calibration curve.

Sample	Estimated *C* (mg/mL)	Estimated *C* (%g/g)
DEF1	478.1 ± 1.4	32.35 ± 0.14
DEF2	477.8 ± 4.8	32.33 ± 0.48

**Table 3:** Estimated mean and uncertainty values of urea concentration in the commercial samples DEF1 and DEF2 using the linear equations reported in Figure 5b as calibration curve.

Sample	Estimated *C* (mg/mL)	Estimated *C* (%g/g)
DEF1	477.3 ± 2.8	32.31 ± 0.28
DEF2	475.8 ± 2.8	32.24 ± 0.28

**Table 4:** Estimated mean and uncertainty values of urea concentration in the commercial samples DEF1 and DEF2 using the linear equation reported in Figure 8 as a calibration curve.

Sample	Estimated *C* (mg/mL)	Estimated *C* (%g/g)
DEF1	473.1 ± 17.3	32.12 ± 1.70
DEF2	493.2 ± 18.0	33.03 ± 1.77

**Table 5:** Summary of the estimated mean and uncertainty values of urea concentration in the commercial samples DEF1 and DEF2, obtained with different methods.

Sample	*C* (%g/g) Photodiode RMS	*C* (%g/g) Photodiode Amplitude	*C* (%g/g) Thermopile Amplitude	*C* (%g/g) HPLC
DEF1	32.35 ± 0.14	32.31 ± 0.28	32.12 ± 1.70	32.89 ± 0.23
DEF2	32.33 ± 0.48	32.24 ± 0.28	33.03 ± 1.77	32.85 ± 0.56

## Data Availability

Data are contained within the article. The original contributions presented in the study are included in the article; further inquiries can be directed to the corresponding author.
